# If self‐shading is so bad, why is there so much? Short shoots reconcile costs and benefits

**DOI:** 10.1111/nph.18636

**Published:** 2022-12-21

**Authors:** Alexandre de Haldat du Lys, Mathieu Millan, Jean‐François Barczi, Yves Caraglio, Guy F. Midgley, Tristan Charles‐Dominique

**Affiliations:** ^1^ AMAP, Univ Montpellier, CIRAD, CNRS, INRAE, IRD F‐34398 Montpellier France; ^2^ Centre for African Ecology, School of Animal, Plant and Environmental Sciences University of the Witwatersrand Private Bag X3 WITS Johannesburg 2050 South Africa; ^3^ Global Change Biology Group, Department of Botany and Zoology Stellenbosch University Private Bag X1 Matieland 7602 South Africa; ^4^ Institute of Botany of the Czech Academy of Sciences v.v.i, Dukelská 135 Třeboň 379 01 Czech Republic; ^5^ CNRS UMR7618 Institute of Ecology and Environmental Sciences Paris, Sorbonne University 4 Place Jussieu 75005 Paris France

**Keywords:** 3D plant modelling, AmapSim, differentiation, plant architecture, self‐shading, short shoot, whole plant, woody plants

## Abstract

- If trees minimize self‐shading, new foliage in shaded parts of the crown should remain minimal. However, many species have abundant foliage on short shoots inside their crown. In this paper, we test the hypothesis that short shoots allow trees to densify their foliage in self‐shaded parts of the crown thanks to reduced costs.
- Using 30 woody species in Mediterranean and tropical biomes, we estimated the contribution of short shoots to total plant foliage, calculated their costs relative to long shoots including wood cost and used 3D plant simulations calibrated with field measurements to quantify their light interception, self‐shading and yield.
- In species with short shoots, leaves on short shoots account for the majority of leaf area. The reduced cost of short stems enables the production of leaf area with 36% less biomass. Simulations show that although short shoots are more self‐shaded, they benefit the plant because they cost less. Lastly, the morphological properties of short shoots have major implications for whole plant architecture.
- Taken together, our results question the validity of only assessing leaf costs to understand leaf economics and call for more integrated observations at the crown scale to understand light capture strategies in woody plants.

## Introduction

With few exceptions, plants perform photosynthesis to meet their carbon requirements. For photosynthesis to be efficient, plants have to display their leaves in a light environment where the carbon gain is positive. A huge body of literature describes how trees optimize their leaf distribution over time (leaf dynamics; Ackerly & Bazzaz, 1995; Kikuzawa, 1995, 2003; Miyazawa & Kikuzawa, 2004; Hikosaka, 2005; Niinemets, 2010) and in space (leaf angle: Kuroiwa, 1970; canopy architecture: Horn, 1971; Honda & Fisher, 1978) for light capture. Because of the considerable number of leaves that make up a tree crown, a certain proportion of the foliage is inevitably shaded by other parts of the crown. Self‐shading reduces the amount of light that reaches the inner part of the crown and also affects its quality (Kitajima *et al*., 2005; Niinemets, 2007; Coops *et al*., 2017). If self‐shading cannot be totally avoided, trees evolved traits to keep self‐shading within a tolerable range, that is above the light compensation point. Moreover, many species are known to develop shade leaves with distinct morphology and physiology (Givnish, 1979, 1988) that can photosynthesize even at intermediate levels of shade and take better advantage of short light pulses through gaps (Chazdon & Pearcy, 1986; Chazdon, 1988). Reducing self‐shading itself can be achieved by either: allowing more light to penetrate the inner parts of the crown, for example through more vertically displayed leaves as in *Eucalypts* (James & Bell, 2000); or by reducing the overlap of leafy layers, for instance through optimization of branch angle (Honda & Fisher, 1978; Pearcy & Yang, 1996; but see Valladares & Brites, 2004); and by adjusting the leaf phenology or pruning excessively self‐shaded leaves (Suzuki & Kohno, 1987). We ask, do self‐shading patterns accord with this idea?

**Fig. 1:**
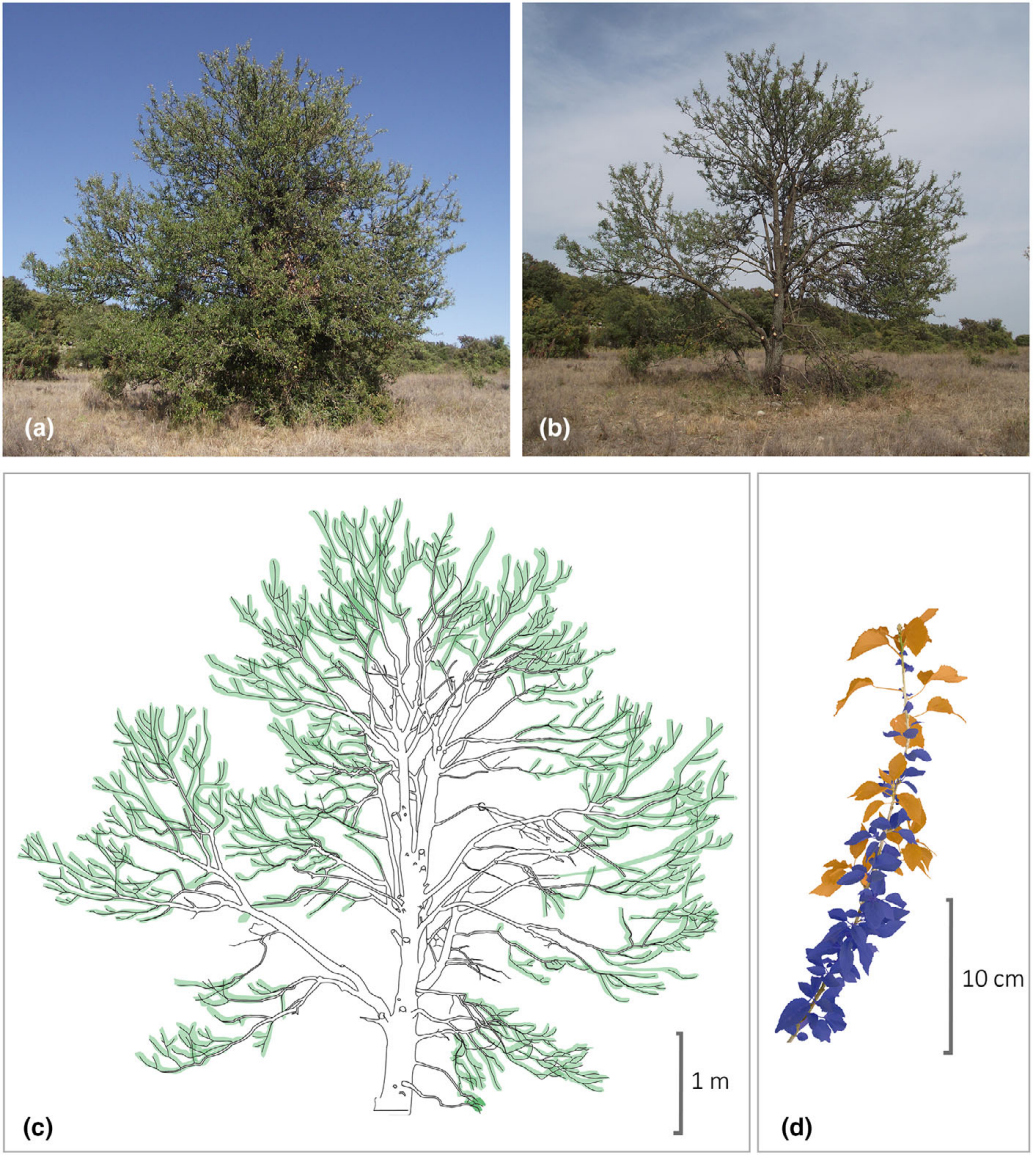
(a) Isolated *Pyrus spinosa* Forss. (Rosaceae) individual growing in an open habitat in a Mediterranean system. (b) We removed half the front section of the crown to examine the thickness of foliage inside. (c) The colour showing the thickness of the foliage (green) of this tree shows that the leaves are not only distributed in a thin peripheral layer but also in a deeper layer, where a large part is in a self‐shaded situation. (d) *Micrococca capensis* (Baill.) Prain (Euphorbiaceae) section exhibiting differentiation in long and short shoots. The leaves supported by the long shoots are coloured orange, and those supported by the short shoots are coloured blue. The elongation of the long shoots vs the shortness of the short shoots separates their foliage. The leaves of the long shoots occupy a peripheral location, while the leaves of the short shoots occupy a more internal location, inevitably more self‐shaded.

As self‐shading is detrimental to trees, they would be expected to locate most of their newly formed leaves in the outermost parts of the canopy. If trees developed entirely to avoid self‐shading, their canopy would resemble an ‘empty shell’, a picture that is quite different to the canopy of most trees in the world that is composed of a rather thick layer of foliage (Fig. 1a–c). A common view of self‐shading is that self‐shaded leaves are mainly leaves that were exposed to the sun in the past and subsequently shaded by newly developed leaves (Ackerly, 1999; Kikuzawa *et al*., 2009). However, architectural descriptions of many tree species rather suggest that most self‐shaded leaves emerge on newly developed stems in areas that are already shaded from either dormant buds (*see sequential delayed reiteration in* Barthélémy & Caraglio, 2007) or highly differentiated stems such as short shoots (*see axis categories in* Barthélémy & Caraglio, 2007). The establishment of short shoots in shaded areas is particularly puzzling under the assumption that trees are optimized to minimize self‐shading. Short shoots are highly differentiated unbranched stems with very little internode elongation and are produced laterally on long shoots, sometimes on very old branches (where the shade is densest). This results in leaves being produced on short shoots inside the crown (Fig. 1d). This raises the question: why do plants produce these morphological structures that suffer from greater self‐shading?

We hypothesize that short shoots are developed in shaded parts of the crown because their lower cost compared with long shoots compensates for their lower exposure to light. The complete costs of displaying a photosynthetic area on a leaf may include not only the primary cost of growing the leaf and its associated stem segment (Barthod & Epron, 2005) but also maintenance costs, hydraulic costs and the biomechanical cost of supporting the additional weight (Poorter, 1994; Givnish, 1995; Terashima *et al*., 2005). While leaf costs have been extensively studied (Givnish, 1979; Kikuzawa, 1991; Eamus & Prichard, 1998; Wright *et al*., 2004, 2005a,b; Poorter & Bongers, 2006; Feng *et al*., 2008), little information is available about the costs associated with stems. In this study, we analysed the primary costs of stems associated with leaf display. We tested the hypothesis that short shoots with extremely reduced internodes are much cheaper to produce than long shoots, and explain why these structures can have a positive carbon balance in self‐shaded parts of the crown (Johnson & Lakso, 1986; Dörken & Stützel, 2009; Dörken, 2012).

First, we evaluated whether the foliage displayed on short shoots corresponds to either a negligible or a major part of the total foliage in 30 woody species in Mediterranean and tropical systems. To this end, we evaluated the proportion of the number of leaves and leaf area of short shoots at the whole crown scale. Second, we analysed the primary costs associated with the leaves and their carrying stems in short and long shoots. Third, we used 3D plant architectural simulated mock‐ups calibrated from field measurements to perform radiative balance simulations and to compare total light capture and the specific yields of short and long shoots. Lastly, we varied the proportion of short shoots, leaf size and stem length individually *in silico* to assess the effects of these morphological variations on light capture and yield. We discuss whether a reduction in gain by short shoots due to self‐shading can be offset by the reduction in cost.

## Materials and Methods

### Study sites and plant material

We conducted our study in two areas with contrasted climates: (1) Hluhluwe–iMfolozi Game Reserve (28°00′S to 28°43′S, 31°70′E to 32°14′E) in KwaZulu Natal, South Africa; and (2) the Montpellier area in the South of France. Both study sites are subject to marked seasonality. The South African site hosts savanna–forest mosaics, while the French site hosts Mediterranean shrubland with riverine forests. We selected 30 deciduous and brevi‐deciduous woody species (Table 1) with long and short shoots (stems with extremely reduced internodes; Barthélémy & Caraglio, 2007; Charles‐Dominique *et al*., 2017) that are dominant in each sampling area (Charles‐Dominique *et al*., 2015). Sixteen species were sampled in South Africa and 14 in France (Table 1). Sampling was performed at the end of the rainy season in South Africa (March–April 2015) and at the end of spring (May–June 2015) in France, when both types of stem (long and short shoots) had completed their growth. We described five mature individuals of each species, giving a total of 150 individuals.

**Table 1:** List of the angiosperm woody species sampled in the study (30 species belonging to nine families), their provenance and leaf habit.

Species	Family	Provenance	Leaf habit
*Acacia gerrardii* Benth.	Fabaceae	South Africa	Deciduous
*Acacia grandicornuta* Gerstner	Fabaceae	South Africa	Deciduous
*Acacia karroo* Hayne	Fabaceae	South Africa	Deciduous
*Acacia nigrescens* Oliv.	Fabaceae	South Africa	Deciduous
*Acacia nilotica* (L.) Willd. ex Delil	Fabaceae	South Africa	Deciduous
*Acacia robusta* Burch.	Fabaceae	South Africa	Deciduous
*Acacia tortilis* (Forssk.) Hayne	Fabaceae	South Africa	Deciduous
*Acer campestre* L.	Sapindaceae	France	Deciduous
*Acer monspessulanum* L.	Sapindaceae	France	Deciduous
*Alnus glutinosa* (L.) Gaertn.	Betulaceae	France	Deciduous
*Amelanchier ovalis* Medik.	Rosaceae	France	Deciduous
*Crataegus monogyna* Jacq.	Rosaceae	France	Deciduous
*Dichrostachys cinerea* (L.) Wight & Arn.	Fabaceae	South Africa	Deciduous
*Gleditsia triacanthos* L.	Fabaceae	France	Deciduous
*Gymnosporia harveyana* Loes.	Celastraceae	South Africa	Brevi‐deciduous
*Gymnosporia nemorosa* (Eckl. & Zeyh.) Szyszyl.	Celastraceae	South Africa	Brevi‐deciduous
*Gymnosporia senegalensis* (Lam.) Loes.	Celastraceae	South Africa	Brevi‐deciduous
*Micrococca capensis* (Baill.) Prain	Euphorbiaceae	South Africa	Deciduous
*Plectroniella armata* (K. Schum.) Robyns	Rubiaceae	South Africa	Deciduous
*Populus alba* L.	Salicaceae	France	Deciduous
*Populus nigra* L.	Salicaceae	France	Deciduous
*Prunus dulcis* (Mill.) D.A. Webb	Rosaceae	France	Deciduous
*Prunus mahaleb* L.	Rosaceae	France	Deciduous
*Prunus spinosa* L.	Rosaceae	France	Deciduous
*Pyracantha coccinea* M. Roem.	Rosaceae	France	Deciduous
*Pyrus spinosa* Forssk.	Rosaceae	France	Deciduous
*Rhus pentheri* Zahlbr.	Anacardiaceae	South Africa	Deciduous
*Robinia pseudoacacia* L.	Fabaceae	France	Deciduous
*Scolopia zeyheri* (Nees) Harv.	Salicaceae	South Africa	Deciduous
*Spirostachys africana* Sond.	Euphorbiaceae	South Africa	Deciduous

### Proportions of shoots in the crown

We first evaluated the proportion of foliage on short shoots. We counted the proportion of long and short shoots in the main subunits that constitute the crown (see ‘total reiterated complexes’; Oldeman, 1974; Barthélémy & Caraglio, 2007). These subunits have an equivalent proportion of short and long shoots and the same organization as the whole crown. We cut one reiterated complex per individual (basal section of *c*. 4 cm and with over 200 stem apices) and counted all the shoots (giving a total of *c*. 1000 shoots per species). We reported the average ratio of short shoots to long shoots for each species. We then analysed the properties of the longest and shortest stems. We counted the number of leaves on two long shoots and two short shoots per individual (i.e. 10 shoots of each type for each species). We then scanned all the leaves (150 d postinoculation) and extracted the total leaf area using ImageJ software. Using the stem count, leaf count per stem and leaf area per shoot, we estimated the contribution of short shoots to the total leaf area of the crown.

### Primary costs of an assimilating area unit

We then evaluated the cost of producing long and short shoots associated with leaf display using their total dry biomass divided by their respective leaf area. We oven‐dried all leaves and stems at 90°C until stable weight was achieved. All costs were evaluated relative to the total leaf area of the shoot. Leaves and carrying stems were weighed separately to the nearest 0.01 g. Leaf costs were analysed using the leaf mass area (LMA) of all the leaves from both types of shoots as described in Perez‐Harguindeguy *et al*. (2013). Stem costs were evaluated as stem dry weight divided by the total leaf area. We compared the costs of displaying the leaf area of long and short shoots using their ratio of total biomass to leaf area.

### Light capture at tree scale and shoot yields

We analysed the incoming light received by all leaves on short and long shoots inside the crown using a 3D virtual model. We used a model to alleviate the difficulty in measuring light accurately in each leaf position without damaging the plant. We first recorded the key morphological variables of the 30 species of all stem types that influence the 3D architecture of woody plants and their leaf display: leaf area, number of leaves, length of stem, number of stems, location of branching, shoot development (monopodial and sympodial), shoot growing direction, phyllotaxy, lifespan of different stem types, etc. We then ranked all the species according to the proportion of short shoots in the crown and selected the three most representative architectural morphotypes, that is the morphotypes representing the largest number of species we analysed that conformed to the architectural properties of the morphotype. The three classes comprised species with a low proportion of short shoots (lower than the first quartile; *n* = 8 spp.), an intermediate proportion of short shoots (between the first and third quartile; *n* = 14 spp.) and a high proportion of short shoots (higher than the third quartile; *n* = 8 spp.).

All simulated morphotypes have deciduous foliage on all their different types of branches. Their phyllotaxy is alternate and the shape of the leaves is unchanged, with a *Prunus armeniaca* leaf shape used as standard. All branching is delayed, and short shoots are pruned after three growth cycles.

**Fig. 2:**
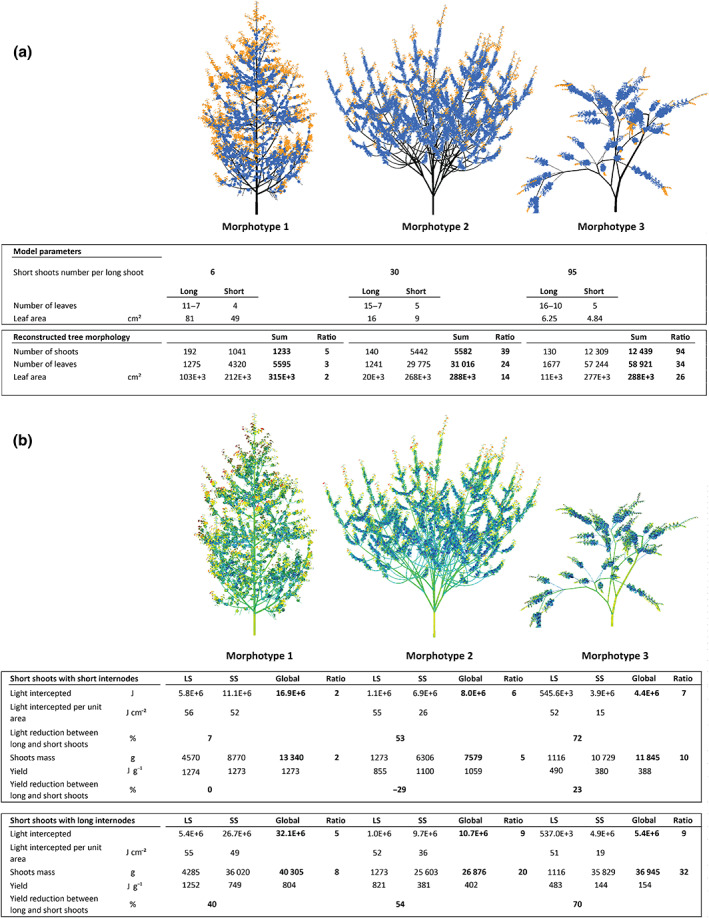
Simulation results. The three morphotypes were reconstructed with architectures matching those of plants with a low, medium and high proportion of short shoots; entries in bold in the tables indicate the global scale values (i.e. the sum of the long and short shoot values), the ratio of the short to long shoot values, and the reductions (in percent) between the long and short shoots. (a) Overview: leaves growing on long shoots are coloured orange; leaves growing on short shoots are coloured blue. At the plant scale, all morphotypes have more shoots, more leaves and bigger leaf area on their short shoots than on their long shoots. (b) Light interception: each leaf is coloured according to its level of light interception (high values are in red and low values in blue; performed by the Archimed‐MIR module; Dauzat *et al*., 2008). A yield was computed for each morphotype (with leaf mass area and wood density set at 0.01 g cm^−2^ and 0.77 g cm^−3^, respectively) as the intercepted light divided by the shoot mass. Short shoots of morphotypes 1, 2 and 3 intercept two, six and seven times more light, respectively, despite the fact less light is intercepted per unit area. In the same way, the total short shoot mass is higher than the total long shoot mass due to the higher number of short shoots. In the lower panel, the internode lengths of short shoots were increased to match those of long shoots. Increasing the length of the internodes of short shoots increases the light intercepted compared with short internodes. This clearly demonstrates the reduction in self‐shading with increasing internode length. On the contrary, increasing internode length also increases shoot biomass. It thus reduces the yield of short shoots and also the total yield of all the morphotypes.

Morphotype 1 has a dominant orthotropic trunk with regularly spaced layers of main branches that are orthotropic and perennial. This organization gives the whole tree a feather‐like shape. Both the trunk and the main branches bear plagiotropic short twigs with long internodes and a shorter lifespan than that of the main stems. Each long stem growth unit shows rhythmic and acrotonic branching. Short shoots are located at the base of the long shoot growth units and on twigs and have a short lifespan. In this morphotype, short shoots bear large leaves (49 cm^2^) but the leaves are produced in low numbers. In the canopy, they account for twice the leaf area, three times the number of leaves and five times the number of shoots than long shoots (Fig. 2a).

Morphotype 2 has a main orthotropic stem that quickly dies and is replaced by multiple basal stems equivalent to the main stem. This organization gives the whole plant a multi‐stemmed shrub habit. Both the trunk and main branches bear orthotropic short twigs with long internodes and a shorter lifespan than the main stems. Each long stem growth shows rhythmic and acrotonic branching. Short shoots are located at the base of the long shoots and on twigs and have a short lifespan. In this morphotype, short shoots have small leaves (9 cm^2^) but are produced in high numbers. They account for 14 times the leaf area, 24 times the number of leaves and 39 times the number of shoots than long shoots in the canopy.

Morphotype 3 has a sympodial structure in which the main stems begin their development as orthotropic and end it plagiotropic (as do most of the *Acacia* species in our study). The main stems have lateral plagiotropic branches and short shoots organized along an acrotonic gradient on each growth unit. Plagiotropic branches also produce short lateral shoots. These short shoots are produced in clusters. In this morphotype, short shoots have minute leaves (4.8 cm^2^) but are produced in a very high numbers. They account for 26 times the leaf area, 34 times the number of leaves and 94 times the number of shoots than long shoots in the canopy.

The three selected morphotypes were then used in simulations to ensure that our simulation conclusions refer to a diversity of background architectures.

We then simulated the 3D architecture of trees to assess the quantity of incoming light on short shoots vs long shoots. We imputed the recorded morphological parameters to develop 3D tree mock‐ups using AmapSim software (Barczi *et al*., 2008). AmapSim is a structural plant model that simulates plant architecture according to a set of architectural rules described in Barthélémy & Caraglio (2007). We parameterized AmapSim to describe the three morphotypes using quantitative variables recorded on plants in the field. The quantitative variables recorded and used to parametrize the model were the length of the shoots, short shoot ratio and the leaf area per shoot in each morphotype (Fig. 2). The three morphotypes were grown virtually in AmapSim until their total leaf area reached a similar value of *c*. 300 000 cm^2^ (the average values for the nine simulated individuals in each morphotype were 313 000 ± 84 000, 287 826 ± 57 000, 287 543 ± 113 000 cm^2^); we then extracted the 3D mock‐ups generated (all with similar total leaf areas but distinct architecture) and computed a radiative balance of all leaves to evaluate the light intercepted by long and short shoots, respectively. We replicated simulations nine times per morphotype to introduce individual variability, as AmapSim can simulate branching and growth parameters with a level of stochasticity. The radiative balance of each of these tree mock‐ups was then extracted at the leaf scale using the Archimed‐MIR module (Dauzat *et al*., 2008). We configured the Archimed‐MIR module to reproduce the solar course from Day 100 to Day 200 of the year at a latitude of 45° with only direct light interception (i.e. no light redistribution after its initial hit on plant structure). MIR computes a light environment with light emitted from 36 directions and reproduces the sun's trajectory each day, as detailed in Dauzat *et al*. (2008). We decided not to include indirect light in our simulations because, in our preliminary analyses, it increased computation time 20‐fold and the required memory space 10‐fold without producing any notable differences in the proportion of light intercepted by long and short shoots. Indirect light provided 7.8% more light to short shoots than to long shoots. The light interception value of each leaf was then extracted using Xplo software (Griffon & De Coligny, 2014) to calculate the light intercepted by long and short shoots. These values were used to quantify the self‐shading experienced by leaves growing on both short and long shoots and compared with shoot dry mass to calculate the yield of each type of shoot. Leaf mass area was set at 0.01 g cm^−2^ and wood density at 0.77 g cm^−3^ (mean values across our species) for both long and short shoots.

We then performed a sensitivity analysis on the 3D model to analyse the effect of several key morphological parameters on the yield of each shoot type. We varied each of the following parameters independently in several successive simulations (totalling 352 simulations): (1) the total proportion of short to long shoots; (2) the size of leaves growing on short shoots; (3) the length of short shoot internodes. All AmapSim parameter files and scripts used in this study are available in Supporting Information (Note S1).

### Statistical analysis

All recorded variables were averaged at the species level (calculated based on five individuals per species). The number of long and short shoots was compared between species using the Wilcoxon Mann–Whitney test for paired data sets, while long and short shoots were compared across species using the Wilcoxon test for independent data. The choice of nonparametric tests was justified by the nonhomoscedasticity of the data. All statistical analyses were performed in R 3.6.2 (R Core Team, 2013).

## Results

### Foliage distribution

**Fig. 3:**
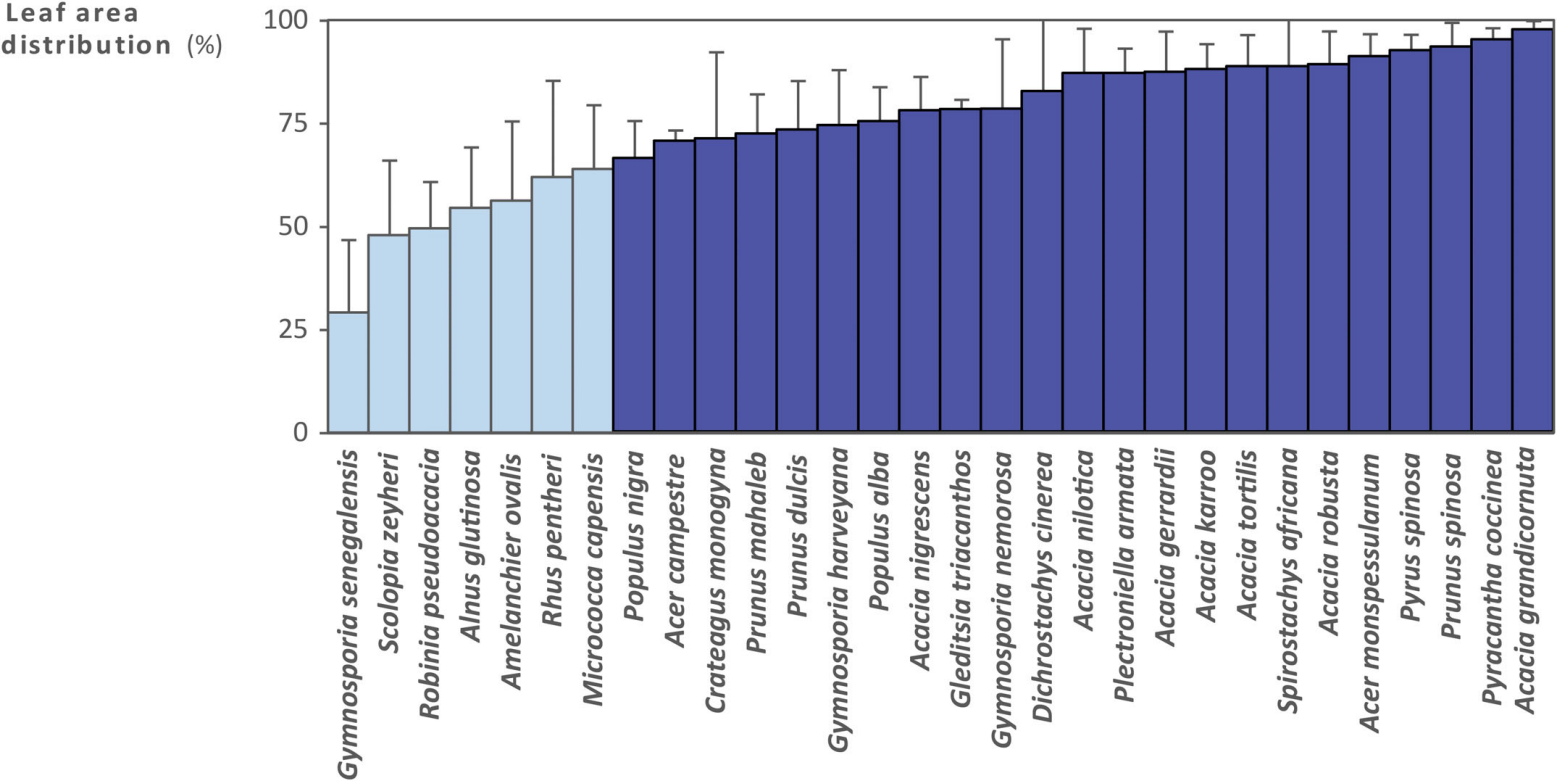
Proportion of leaf area at tree scale on short shoots. Error bars represent SDs. Dark blue bars indicate species with a significantly greater leaf area supported by short shoots than that supported by long shoots (*P* < 0.05, *n* = 5, Wilcoxon Mann–Whitney, unilateral).

In all the species recorded, short shoots were the most abundant shoot types in the crown (Wilcoxon Mann–Whitney tests; all **, *P* < 0.01; *n* = 5) and accounted for the largest proportion of foliage. The average was 41 ± 39 short shoots per long shoot (Fig. S1). Although short shoots were clearly more abundant than long shoots (Fig. S1), marked variability was observed between some species with a low ratio including *Gymnosporia senegalensis* and *Robinia pseudoacacia*, both of which had 4 ± 2 short shoots per long shoot, and species with a high ratio including *Acacia grandicornuta*, which had 164 ± 73 short shoots per long shoot. For each species, the leaf area associated with one stem was significantly higher for long shoots than short shoots (Wilcoxon Mann–Whitney test; **, *P* < 0.01; *n* = 5). On average, short shoots had 6 ± 4 times less assimilating area than long shoots. This difference is mainly explained by the fact there were three times more leaves on long shoots (14.0 ± 5.1 leaves) than on short shoots (4.7 ± 1.5 leaves). Leaves growing on long shoots were also generally larger than leaves growing on short shoots but the difference was only significant in 14 cases (*, *P* < 0.05; *n* = 5; Table S2, see later). At the crown scale, the number of leaves growing on short shoots was significantly higher in 29 of the 30 species studied (*, *P* < 0.05; *n* = 5) and accounted for an average of 83 ± 8% of crown leaves. The leaf area represented by short shoots was significantly greater in 23 out of the 30 species (*, *P* < 0.05; *n* = 5; Fig. 3). Across all species, short shoots accounted for an average of 74 ± 11% of the total leaf area. In *Alnus glutinosa*, *Amelanchier ovalis*, *Rhus pentheri*, *R. pseudoacacia*, *Scolopia zeyheri* and *Micrococca capensis*, the leaf area represented by short shoots did not differ significantly from that represented by long shoots. Only *G. senegalensis* had significantly more leaves and a bigger leaf area on long shoots (*, *P* < 0.05; *n* = 5; Wilcoxon Mann–Whitney). Short shoots of *morphotype 1*, *morphotype 2* and *morphotype 3* accounted for, respectively, 59 ± 15%, 76 ± 12% and 91 ± 4% of the leaf area at tree scale and for 86%, 97% and 99% of the total number of stems (Fig. S1).

### Primary cost of setting up leaf area

**Fig. 4:**
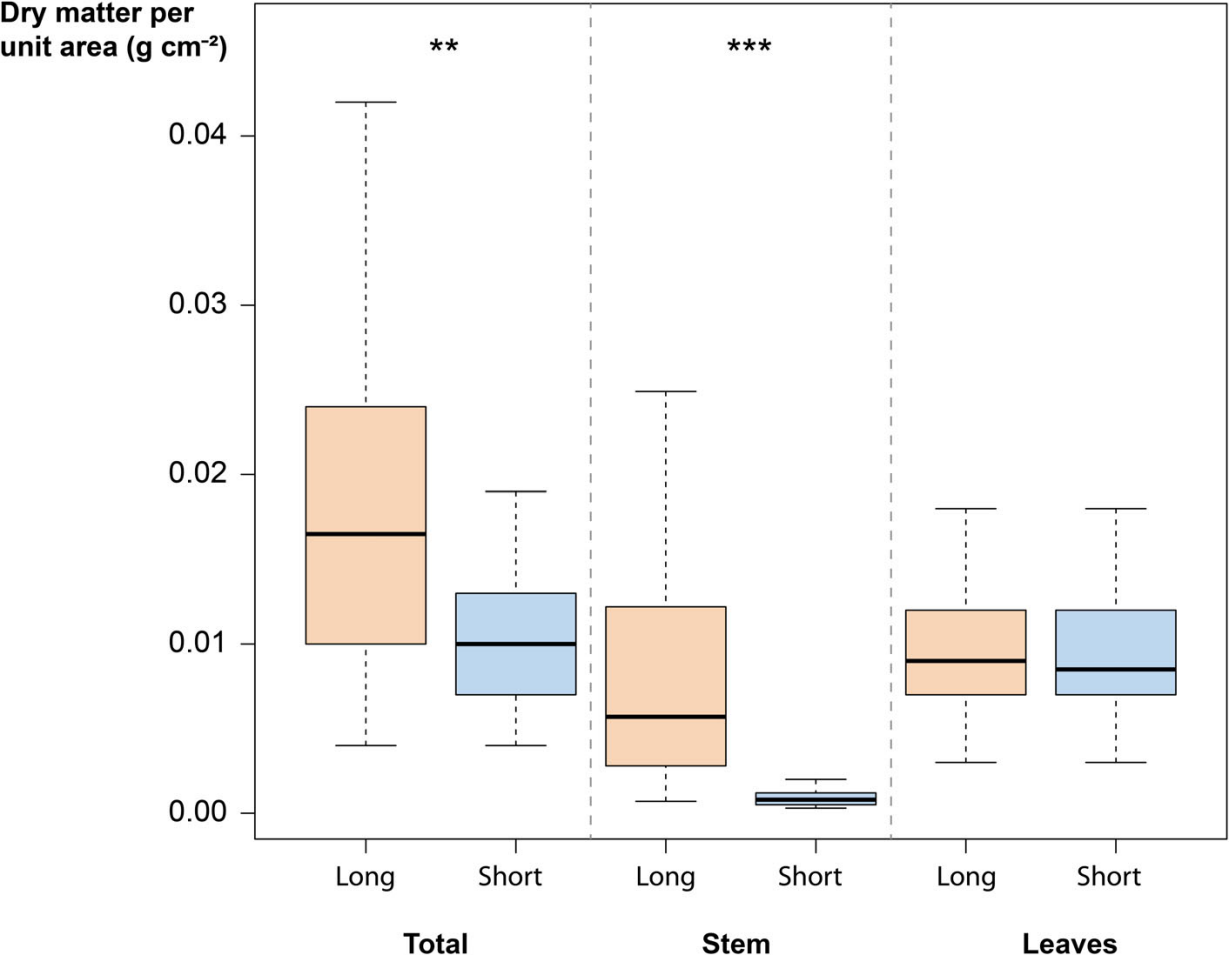
Over all the species sampled, dry matter required to set up an assimilating area unit on long shoots (in orange) and short shoots (in blue). The total cost is calculated as the ratio of dry mass of the leafy shoot to its assimilating area. We broke down the total cost into the cost of the carrying stem (corresponding to the ratio of dry mass of the annual stem production to its assimilating area) and the cost of leaves (corresponding to the ratio of dry mass of the shoot leaves to its assimilating area). The reduction in the cost of the foliage of short shoots is linked to a reduction in the cost of the carrying stem. The whiskers correspond to the first and ninth decile, the lower and upper hinges of the boxplot correspond to the first and third quartile, the black line inside the box marks the median. Asterisks indicate whether the means comparison test was found to be significantly different between the cost of long and short shoots: **, *P* < 0.005; ***, *P* < 0.0005 (*n* = 30; Wilcoxon, bilateral).

The primary costs associated with leaf area are higher on long shoots than on short shoots due to a difference in stem costs associated with leaf production but not with the leaf costs *per se*. The total cost of a similar leaf area on short shoots was 36 ± 17% lower than on long shoots. The reduction in costs was 22 ± 10% for species with a lower proportion of short shoots in their crown (morphotype 1), was 37 ± 17% for species with an intermediate proportion of short shoots in their crown (morphotype 2) and was 47 ± 14% for species with a higher proportion of short shoots in their crown (morphotype 3). The reduction in primary costs associated with establishing a given leaf area depends on a reduction in stem biomass per leaf area, which was significantly higher for long shoots than for short (paired Wilcoxon Mann–Whitney; ***, *P* < 0.005; *n* = 30), while no significant difference was found in the LMA of long and short shoots (paired Wilcoxon Mann–Whitney; not significant; *n* = 30; Fig. 4). The stem costs associated with a similar unit of leaf area were on average 12.1 ± 11.6 greater for long shoots than for short.

### Light interception and yield

Simulated short shoot leaves produce a better yield than long shoots after all their primary production costs are taken into account. In our simulation, the leaf area of short shoots is 2, 14 and 26 times greater than the leaf area of the long shoots, respectively, for morphotypes 1, 2 and 3 (Fig. 2a). All simulated trees have significantly more biomass on short shoots than on long shoots (Fig. 2a). After computing the radiative balances (Fig. 2b), peripheral leaves intercept more light than leaves located inside the crown. Trees with a higher proportion of short shoots have more self‐shading, as they intercept less total light than trees with a smaller proportion of short shoots (for an equivalent total leaf area; Fig. 2). Overall, short shoots of morphotype 1, 2 and 3 models intercept, respectively, two, six and seven times more light than long shoots. Leaves on short shoots are on average more self‐shaded with a lower light interception per leaf area than long shoots (Fig. 2b). Leaves on short shoots of morphotypes 1, 2 and 3 receive on average, respectively, 7%, 53% and 72% less light than leaves on long shoots. The reduction in yield from long to short shoots on morphotype 3 is 23%, while for morphotype 2, an increase of 29% was observed for short shoots (Fig. 2b), and morphotype 1 shows equal yields for long and short shoots.

In our simulated trees, the whole plant yield is maximized according to the number of short shoots and their morphology. Plants with relatively fewer short shoots (morphotype 1) have a better yield with less differentiated short shoots (more similar to long shoots with large leaves and long internodes; Table S1). On the contrary, plants with more short shoots (morphotype 3) have a better yield only if the short shoots are highly differentiated (with different morphological parameters than long shoots, that is with shorter internodes and smaller leaves; Table S1). When we artificially varied the morphology of short shoots moving away from these observed rules (number of short shoots per long shoot, length of internodes and leaf size), the overall yield of plants decreased (Table S1). Several simulation outputs indicate that the overall light capture can increase, while the overall yield decreases, showing that less self‐shading is not always beneficial in terms of yield. For example, when we reduced the internode length of short shoots, the light captured by the whole plant light decreased but the yield increased as it was associated with an overall reduction in cost. As a summary, plant yield would be lower if short shoots were longer, more numerous or their leaf size bigger.

## Discussion

### Plant leaves do not float in thin air

To be produced, all leaves require a carrying stem and our results suggest that the properties of the carrying stem have very strong implications for the cost of leaf production, their location in the crown (with consequences for their light environment) and the resulting density of the foliage. In this paper, we analyse how the foliage is distributed between short and long shoots in 30 woody species belonging to nine families in two different biomes, one in South Africa and one in the South of France. We evaluated the relative contribution of each type of shoots in terms of biomass and light interception in simulations. We found that, for species with short shoots: the great majority of leaves grow on short shoots and short shoots produce a high proportion of the photosynthetic area; leaves growing on short shoots on average grow in more self‐shaded area; short shoots can however be beneficial in shaded area as they have a lower production cost mainly due to the reduced costs of their stems compared with those of long shoots; and the dimensions of short shoots have consequences for their proportion in the crown: Species with shorter internodes on their short shoots produce a higher proportion of short shoots in the crown. These considerations question the validity of standard protocols that focus on measurements taken on long shoots to understand the ability of a species to capture light efficiently, and call for assessing the costs of assimilating organs by including all related costs (not only organ production costs but also all related architectural costs). This study shows that results obtained for isolated organs differ depending on where they are collected from the whole plant. It suggests that the structure of the axes (and not only organ properties) should be included in analyses of plant assimilation strategies. Later, we discuss these points one by one and propose possible ways to better understand plant functioning by accounting for the differentiation of shoot types.

### Short shoots are frequently overlooked when plant functioning is analysed even though they may play the main role in key functions such as light capture

The standard protocol handbook even excludes short shoots by recommending that all leaf traits be recorded on well‐developed shoots with long internodes exposed to full sun (Cornelissen *et al*., 2003; Perez‐Harguindeguy *et al*., 2013). In most physiological studies, leaf properties are recorded with no mention of the type of stem that bears them. For plants with a strong stem differentiation (difference in morphological properties between stem types), our results confirm those of previous studies (Wilson, 1966, 1991; Jones & Harper, 1987; Charles‐Dominique *et al*., 2012; Dörken, 2012) showing that each long shoot is associated with a very high number of short shoots (41 short shoots per one short shoot on average in our species). We further found that short shoots bear, on average, 83% of the leaves and account for 74% of the leaf area. While relative light interception by long shoots and short shoots has never been quantified due to the huge number of leaves in the crown of woody plants, several authors posited that short shoots that are produced laterally on long shoots should on average, be more internal in the crown and therefore more self‐shaded (Titman & Wetmore, 1955; Powell, 1988; Sabatier & Barthélémy, 1999; Yoshimura, 2010; Dörken, 2012 and references within). Interestingly, the leaves displayed on short shoots are not merely self‐shaded due to the subsequent development of a more external layer of leaves on long shoots, but are rather actively developed in shaded areas. Several properties of short shoots contribute to increasing self‐shading: their leaves are located inside the crown as the branching of short shoots is delayed compared with that of long shoots; short shoots have limited exploration capacity due to their short internodes, which prevents them positioning their leaves farther away from their initial emission point; short shoots are frequently produced at the same location several years in a row either by having a pluriannual lifespan or by branching that occurs close to their insertion point (from accessory buds or sprouting from their base). The high proportion of foliage displayed on short shoots that are *a priori* more self‐shaded than long shoots is puzzling, as it strongly contradicts a general hypothesis that plants are organized to display leaves in positions that minimize self‐shading (Horn, 1971; Honda & Fisher, 1978; Fisher & Honda, 1979; Ackerly & Bazzaz, 1995; Kikuzawa, 1995). We performed simulations to further analyse whether or not short shoots leaves are indeed more self‐shaded.

The results of our simulations, which were calibrated using real plant measurements, confirm that leaves on short shoots are located in areas that are more self‐shaded than long shoots. This reduction in incoming light is not offset by leaf construction costs. After building realistic 3D plant mock‐ups using architectural parameters recorded in 30 woody species, we quantified the incoming light on each leaf and calculated the whole plant radiative balance (a total of 378 plants simulated). We found that leaves growing on short shoots receive 7%, 53% and 72% (on average) less light per unit area than leaves growing on long shoots, for, respectively, morphotypes 1, 2 and 3. When calculating the production costs of the leaves (approximated by their biomass per area), we found no difference between long shoots and short shoots, in agreement with Miyazawa & Kikuzawa (2004). The reduced incoming light on the leaves growing on short shoots does not appear to be explained by a reduction in the cost of the leaves. In light of these results, the very high production of short shoots could be beneficial for the plant if one of the following conditions is met: leaves growing on short shoots with the same biomass per area could function as shade leaves with a different physiology that is adapted to shaded conditions (Givnish, 1988; Dörken & Lepetit, 2018). To our knowledge, only the study by Dang Le *et al*. (2013) explicitly addresses whether differences in leaf anatomy or physiology are explained by distribution according to shoot types vs growing in full light or in shade. Dang Le *et al*. (2013) found that the type of shoot has a much stronger effect on leaf anatomy than their light environment, but the question remains to be investigated across large pools of species. Likewise, the physiological properties, and more specifically the ability of leaves growing on long and short shoots to perform photosynthesis at low light levels, require further investigation to understand whether physiological adjustments also occur between shoot types; (2) leaves in a slightly shaded environment could perform better photosynthesis as they are less exposed to excessive light and temperatures at mid‐day, especially during periods of water deficit (Givnish, 1984; King, 1997; Valladares & Pearcy, 1998; Schieving & Poorter, 1999) and to less risk of damage caused by environmental factors (Valladares & Pugnaire, 1999); and (3) all the costs associated with developing leaf area are not well described by the cost of production of the leaf organ. We thus further investigated whether the costs associated with the establishment of a leaf on short shoots are lower than the costs of establishing a leaf on long shoots.

Integrating the primary stem costs associated with leaf production revealed that the overall yield of short shoots is higher than that of long shoots. In short shoots, the lower stem cost compensates for their lower exposure to light. While the biomasses per area are equivalent for long shoots and short shoots, the portion of long stem associated with each leaf (internode) is much larger for long shoots than short shoots, in agreement with Dörken's suggestion (2012). Our results indicate that the primary cost of an equivalent leaf area on short shoots is 36% lower than on long shoots. Our simulations show that the reduction in costs associated with stems is high enough to compensate for the reduction in light availability experienced by the leaves on short shoots due to their location inside the plant crown and self‐shading pattern. We also performed a virtual experiment in which we transformed all short shoots on a plant into long shoots to evaluate how this translates into light capture and resulting cost–benefit (approximated by a ratio of biomass to light intercepted). In this experiment, we applied the morphological characters of the long shoots to all the short shoots. After having artificially elongated short shoots, we found that the overall self‐shading in the plant decreased but the overall cost–benefit ratio increased. In other words, short shoots are only profitable because of their short internodes, even if this increases self‐shading. An important caveat in terms of quantification, but not for the conclusion of our study, is that we only recorded leaf and stem costs associated with their primary growth (and approximated by biomass). The other aboveground costs associated with the display of a leaf area that were not quantified include costs of (Barthod & Epron, 2005): leaf respiration; leaf maintenance; respiration and maintenance of all stem tissues resulting from the addition of the leaf; mechanical support on the stem associated with the addition of the leaf weight; and anatomical structures to allow sap flows from the leaf to the root system. Belowground, these costs should ideally be complemented by root costs (Eissenstat, 1992). Accurately quantifying (and therefore simulating) all these costs was beyond the scope of our study, but because short shoots are lighter per leaf area, their exported biomechanical costs on the stem should be reduced; their more internal location should require less investment in hydraulic architecture (Givnish, 1984); finally, their extremely reduced volume should result in lower costs associated with respiration and maintenance. Lastly, the anatomical composition of short shoots with very reduced wood production also reduces their costs (Little *et al*., 2013).

Understanding light capture requires observations at the whole crown scale, as the whole crown strategy depends to a great extent on differentiation in the type and number of stems. Short shoots can easily be identified within a species, as they usually have very stable morphological properties compared with other categories of leafy shoots (Yagi, 2000). However, by comparing multiple species, we showed that a large gradient of more or less differentiated short shoots exists, that is morphological properties that differ to varying degrees from those of long shoots (Table S2). Species with the highest proportions of short shoots have smaller leaves on both long and short shoots and have lighter short shoots than species with fewer short shoots. We found that species with ‘cheap’ short shoots (with extremely reduced construction cost per leaf unit area) produce more short shoots in the crown. This means that when comparing species with low‐to‐high specialization of short shoots for light capture (with lower associated construction costs), the light is increasingly captured by short shoots leaves. Furthermore, Dörken (2012) found that deciduous species frequently have highly differentiated short shoots. The differentiation of short shoots therefore has important consequences for crown composition and probably strongly impacts their ecological performance in different light environments (Margolis *et al*., 1995; Van Pelt & Franklin, 2000; Hirose, 2004). Furthermore, our observations suggest that the level of differentiation of short shoots may be linked to the crown architecture. Even though the number of species was too small for us to draw general conclusions, species with a low, medium and high ratio of short shoots in their crown had very different dominant morphotypes, which we used to represent architectural variability in our simulations (Fig. 2). Further studies are required to analyse how the differentiation of short shoots impacts the plant economic spectrum and its whole organization. These results also have implications for better understanding the role of light capture with respect to competitive interactions between plants. For example, our results would predict a higher proportion of short shoots in plants that prioritize suppression of competitors through light deprivation. Additionally, it is likely that the timing of establishing leaves on different types of shoots differs and affects their relative benefits. In some species, the foliage on short shoots can be produced before that on long shoots, thereby reducing their total self‐shading and giving them a greater carbon gain during early regrowth, especially after a disturbance (Palacio *et al*., 2011).

In this study, we have shown that the morphological differentiation of short shoots greatly reduces the costs of producing leaves and could explain why some plants can tolerate greater self‐shading. However, the effect of stem specialization on their function is not only limited to reducing the cost of light capture but also influences many other functions, as reported in previous studies; for example, many species preferentially develop flowers and fruits on short shoots (Wilson, 1966; Barthélémy & Caraglio, 2007; Costes *et al*., 2014); short shoots could also be used to display foliage in positions that are already protected from herbivores by spines or cagey architecture (Charles‐Dominique *et al*., 2017). Lastly, it is important to note that short shoots represent the most differentiated shoot types compared with the main shoots but that most woody species with short shoots also have intermediate categories of axes (Barthélémy & Caraglio, 2007) that are – for example – important for multiplying the number of short shoots in the crown. We suggest that, as exemplified here by the differences between long and short shoots, many (if not all) plant functions are strongly partitioned according to the plant shoot types. This partitioning of functions across shoot types probably differs considerably across ecosystems with different dominant constraints and could thus play a key role in explaining species ecological performance.

#### Limitations and future outlook

The aim of the present study was to compare the additional costs and benefits of having short shoots in a more self‐shaded location. Of course, other factors affect the benefits and costs of the different types of shoot that compose the canopy. Among the factors we did not include, mostly due to technical limitations, several call for further research to understand the role of short and long shoots in capturing light: the properties and physiological performances of leaves; the indirect costs associated with different types of shoot; the spatial arrangement of their leaves; and their relative phenology.

1. In this study, we kept all parameters associated with leaves constant, except leaf size which was parameterized using field measurements. However, several leaf morphological parameters have been shown to affect light interception, including leaf inclination, leaf shape and phyllotaxy (Valladares & Brites, 2004; Strauss *et al*., 2020). In addition, daily movements due to the opening and closing of the blades according to the light level also influence overall light interception (Liu *et al*., 2007). In the present study, we considered light interception at the canopy scale. To extend the study to photosynthesis, it will be important to recall that the photosynthetic response of leaves to light is not linear and that other factors need to be taken into account. In full light, leaf light saturation may change the radiative balance at certain periods of the day and according to the light environment (cloudiness, temperature, CO_2_ availability, evaporation in full light vs in the shade, etc.). Likewise, leaves exposed to the sun and leaves shaded by the canopy could have a distinct photosynthetic response to the same received light (Givnish, 1988). Further investigation of these parameters on the different shoot types is thus recommended.
2. The costs addressed in this study only incorporate the primary costs related to leaf establishment and their associated internodes. To get a more holistic evaluation of costs, other costs should be taken into account, such as the hydraulic and mechanical costs that are paid on older branches, the trunk and roots connecting these leaves to the parts of the root system responsible for nutrient uptake, and the maintenance costs paid for each type of shoot type.
3. We intentionally reduced the complexity of plant architecture from 30 different species into three morphotypes to keep the analyses simple and focussed on our research question, but the diversity of architectures and resulting tree shapes requires further analyses as they profoundly influence the rules of light acquisition. Among the parameters to investigate as a priority would be the number and properties of the types of shoot that are intermediate between long shoots and short shoots, as they affect the arrangement of leaves in the canopy and could exponentially increase the number of short shoots in the crown.
4. Last, as discussed previously, the phenology of the leaves on each type of shoot could differ. Future studies should investigate how this differential phenology affects the radiative balance during development and sum up at the whole plant scale.

## Competing interests

None declared.

## Author contributions

AHL, MM, J‐FB, YC and TC‐D conceived the project. AHL and TC‐D collected and analysed the data. AHL and J‐FB conceived and performed simulations. AHL, J‐FB and TC‐D wrote the first draft of the manuscript. All other authors contributed substantially to revisions.

## Supporting information


**Fig. S1** Number of short shoots per long shoot in the 30 woody species sampled.

**Table S1** Light interception by simulated mock‐ups and related costs.

**Table S2** Average specific values for each shoot type (short and long shoots) of five morphological variables.

**Note S1** Compressed archive containing all AMAPSIM parameter files and scripts of the simulations performed.
Please note: Wiley is not responsible for the content or functionality of any Supporting Information supplied by the authors. Any queries (other than missing material) should be directed to the *New Phytologist* Central Office.

## Data Availability

The data that support the findings of this study are openly available in Dryad Data Repository at doi: 10.5061/dryad.79cnp5hzh.
