# Supplementary material for: If self‐shading is so bad, why is there so much? Short shoots reconcile costs and benefits
Source: New Phytol. 2022 Dec 21;237(5):1684–95. doi: 10.1111/nph.18636 (PMC10107860; doi:10.1111/nph.18636)
Supplement: Supplementary file 1 — Fig. S1 Number of short shoots per long shoot in the 30 woody species sampled. [file NPH-237-1684-s003.pdf]

New Phytologist Supporting Information

Article title: If self-shading is so bad, why is there so much? Short shoots reconcile costs and benefits.

Authors: Alexandre de Haldat du Lys, Mathieu Millan, Jean-François Barczi, Yves Caraglio, Guy F. Midgley, Tristan Charles-Dominique.

Article acceptance date: 19 November 2022

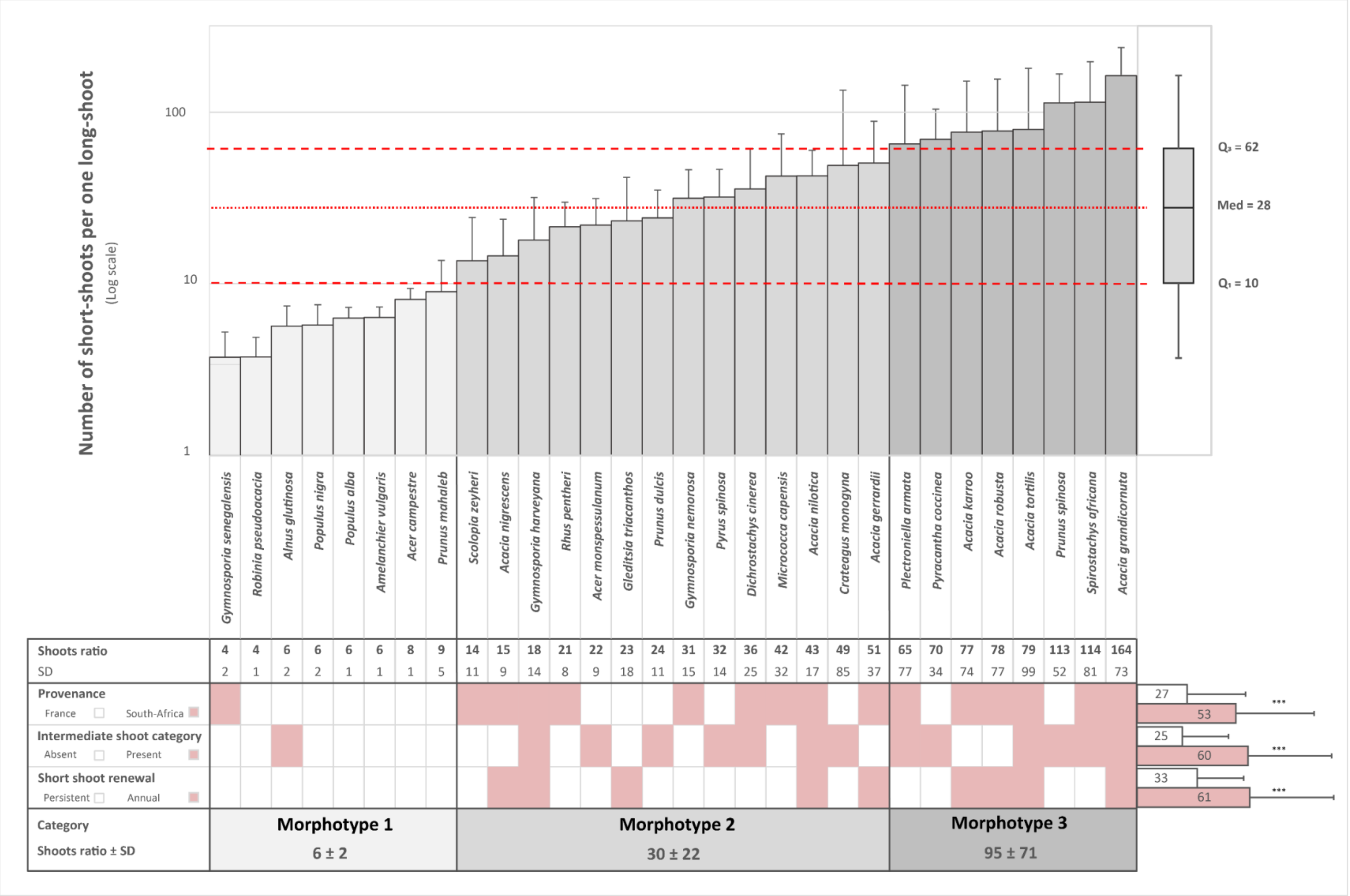

Fig. S1 Number of short shoots per long shoot in the 30 woody species sampled (logarithmic scale). The median (Med) is 28 short shoots per one long shoot. The species are divided into three classes, morphotype 1, 2 and 3, according to their quartile ranking. There are 8 species lower than the first quartile (Q1), 14 species between the first and third quartile (Q3), and 8 species over the third quartile Q3. These categories are subsequently used for the calibration of morphotypes. The effects of the provenance (France vs South Africa), the setting-up of an intermediate shoot category between long and short shoots (absent vs present), and the type of renewal of short shoots (annual vs perennial) are tested (Wilcoxon test for independent data, *** P < 0.005). The ratio of short shoots per long shoot of these different modalities is shown in the histograms at bottom right. Error bars represent the standard deviation.
