# Supplementary material for: If self‐shading is so bad, why is there so much? Short shoots reconcile costs and benefits
Source: New Phytol. 2022 Dec 21;237(5):1684–95. doi: 10.1111/nph.18636 (PMC10107860; doi:10.1111/nph.18636)
Supplement: Supplementary file 4 — Note S1 Compressed archive containing all AMAPSIM parameter files and scripts of the simulations performed. Please note: Wiley is not responsible for the content or functionality of any Supporting Information supplied by the authors. Any queries (other than missing material) should be directed to the New Phytologist Central Office. [file NPH-237-1684-s002.file › supplementaryMaterial/HOWTO.pdf]

How to run simulations and extractions ?

1) download and install Amapsim (https://amapstudio.cirad.fr/soft/vitis/running) and Xplo (https://amapstudio.cirad.fr/doku.php?id=download)

2) copy the parameter files (files with fpa, cfg, dta, fca, ops extension) into a local directory. Create a "opf" directory into this local directory.

3) copy every computing scripts (files with the cmd extension) and extraction scripts (files with a groovy extension) to the same local directory

4) edit every cmd and groovy files so the paths match your configuration

5) run every computing scripts to generate 3D mockups.

6) run extraction groovy script into xplo.

In case of problems, contact barczi@cirad.fr
