# Supplementary figures and images for: If self‐shading is so bad, why is there so much? Short shoots reconcile costs and benefits
Source: New Phytol. 2022 Dec 21;237(5):1684–95. doi: 10.1111/nph.18636 (PMC10107860; doi:10.1111/nph.18636)

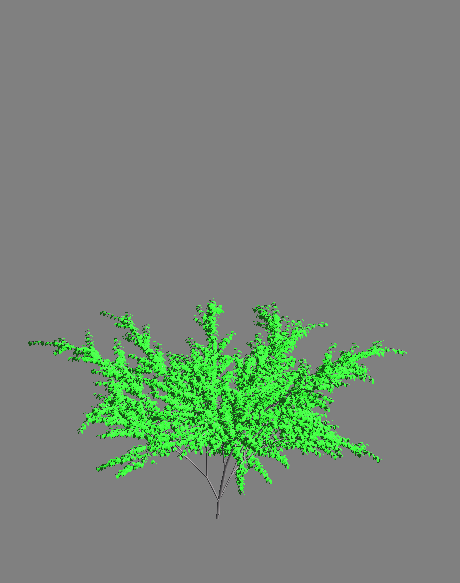

Supplement: Supplementary file 4 — Note S1 Compressed archive containing all AMAPSIM parameter files and scripts of the simulations performed. Please note: Wiley is not responsible for the content or functionality of any Supporting Information supplied by the authors. Any queries (other than missing material) should be directed to the New Phytologist Central Office. [file NPH-237-1684-s002.file › supplementaryMaterial/quartile3Troll.png]
